# FasterNet-YOLO for real-time detection of steel surface defects algorithm

**DOI:** 10.1371/journal.pone.0323248

**Published:** 2025-05-08

**Authors:** Shiwei Yu, Zelin Liu, Liang Zhang, Xiaoqiang Zhang, Jikui Wang

**Affiliations:** CGN Digital Technology Co., Ltd., Shanghai, China; University of Exeter, UNITED KINGDOM OF GREAT BRITAIN AND NORTHERN IRELAND

## Abstract

Steel surface defect detection is an important application of object detection in industry. Achieving object detection in industry while balancing detection accuracy and real-time performance is a challenge. Therefore, this paper proposes an improved FasterNet-YOLO model based on the one-stage detector. Introduce the FasterNet network to reconstruct the YOLOv5 backbone network. Achievement of model lightweighting and significant improvement in detection speed, but with a slight reduction in accuracy. The YOLOv5 neck network’s ordinary convolution is improved by depthwise separable convolution. Continuing to improve detection speed while further reducing redundant parameters in the neck network. To improve model accuracy, the Swin-Transformer is integrated into the C3 module in the neck network. Solve the problem of cluttered backgrounds in defect photographs and easy confusion between defect types. Meanwhile, BiFPN is used for feature fusion. By retaining more informative features, the detector’s ability to adapt to targets at different scales is improved. The results indicated that when comparing FasterNet-YOLO with the original model, the parameters were reduced by 49.4%, GFLOPs were reduced by 57.0%, mAP increased by 6.2%, and FPS increased by 54.1%. The improved model not only increases the detection accuracy, but also significantly improves the speed of hot-rolled strip surface defect detection to meet the requirements of real-time detection.

## 1 Introduction

Several factors can influence the production process, including continuous casting of steel billets, processing techniques, and the production environment. Steel surfaces typically have defects such as crazing, inclusion, patches, pitted surfaces, rolled-in scale, and scratches [1]. These defects not only affect the appearance, but also lead to stress concentrations. As a result, the steel’s capacity to tolerate fatigue and collisions is reduced, limiting its lifespan. The manufacturing of defective steel wastes a significant amount of raw materials, which has a negative impact on the enterprise’s efficiency. The current urgent issue is to solve the problem of controlling defective steel productivity and improving product quality to meet the requirements of modern industry. Therefore, it is crucial to improve the ability to detect surface defects in steel [2].

Traditional steel surface defect detection methods are classified into three categories: manual detection, photoelectric detection and traditional machine vision-based detection methods [3–5]. Manual testing method is inefficient and labor-intensive, with issues such as inconsistent testing standards and missing tests. Despite its increased effectiveness, the photoelectric detection approach is limited by strict environmental conditions and requires significant equipment maintenance expenses [6]. Traditional machine vision-based inspection approaches have significantly enhanced the speed and precision of inspections. However, they still require manual segmentation and feature extraction from steel photos. The operator must have a high level of technical expertise, and the computer must have significant computing power. With the rapid advancement of artificial intelligence, particularly deep learning technology, has provided a more efficient approach for detecting defects on steel surfaces in recent years [7,8].

Defect detection approaches based on conventional machine vision are divided into several processes, including image preprocessing, threshold segmentation, feature extraction, and classifier classification. Threshold segmentation and feature extraction are difficult procedures that require manual design [9–12]. With the increase of industrial activities, the number of data generated in actual production is continuously increasing, which is leading to greater demands for the performance of algorithms [13–15]. In 2014, Girshick et al. proposed the R-CNN algorithm based on Convolutional Neural Networks (CNN) [16]. The VOC2007 dataset achieved a mean Average Precision (mAP) of 58.5%, representing a significant gain compared to the old algorithm’s 33.7%. Presently, the majority of steel surface defect detection models that utilize deep learning are developed using the Faster RCNN model, YOLO series model, and SSD series model [17–22]. Each of the three designs has distinct principles and focuses on specific performance aspects. The Faster RCNN model is based on the two-stage network architecture of R-CNN. The design approach involves the use of region proposal, CNN, and Support Vector Machine (SVM). The CNN network is used to extract features. The region proposal algorithm is used to generate candidate regions and correct the coordinates. The final classification and localization results are obtained through full connectivity and classifiers. Wang M et al. proposed an image detection method based on improved Faster R-CNN model for wear location and wear mechanism identification [23]. The YOLO series and SSD series networks are categorized as single-stage network structures and employ a single-stage design concept. This class of network avoids the need for adjusting the candidate box and can directly get the category probability and position information of the target. While there is a trade-off in terms of precision, the speed of detection is significantly improved. Li Z et al. proposed a lightweight and efficient deep learning model (SSDD-Net) for steel surface defect detection. The proposed model obtains optimal performance while keeping a small number of parameters (3.79M) [24]. Zhao C et al. proposed a model, named RDD-YOLO, based on YOLOv5 for steel surface defect detection, a higher level of precision is achieved [25].

In order to solve the problems of slow detection speed, low accuracy, and challenging deployment of low arithmetic power equipment in hot rolled strip steel surface defect detection model. The FasterNet-YOLO hot-rolled strip surface defect detection model is proposed. The model allows for rapid and exact automatic identification of surface defects on hot-rolled strip using low computational power devices.

## 2 Proposed methods

**Fig 1:**
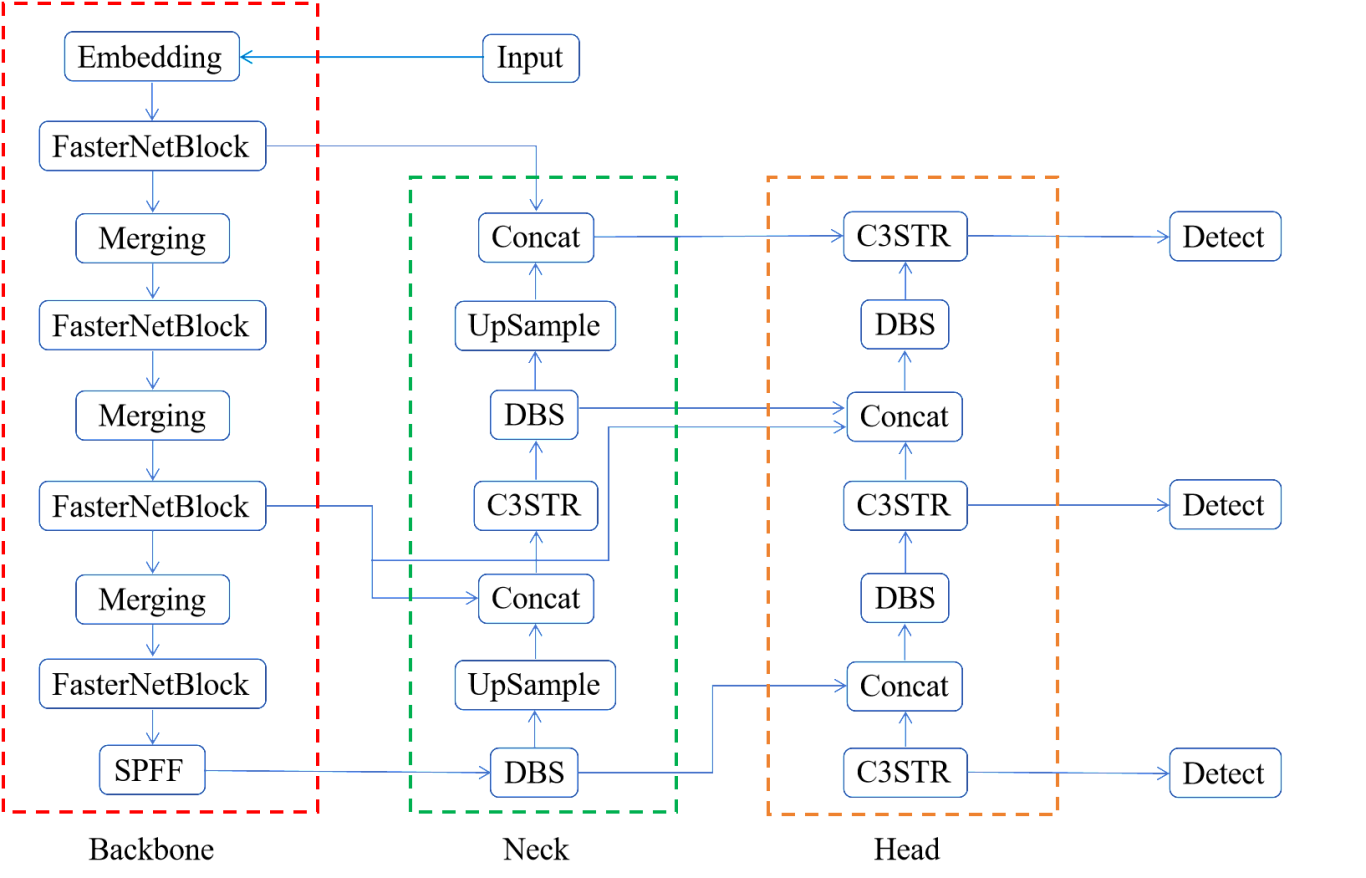
The network structure of FasterNet--YOLO.

YOLOv5 is a deep learning based target detection model. The model takes the entire image as input and directly predicts the bounding box and category probabilities of the target within the image. YOLOv5 has received much attention for its robustness, generalization ability, fast detection speed and high accuracy. In this paper, we propose a FasterNet - YOLO lightweight surface defect detection model for low-computing power devices based on the YOLOv5 model. The network structure of FasterNet--YOLO is shown in Fig 1.

FasterNet - YOLO is mainly composed of three parts: the backbone network, the neck network and the detection head. The backbone network extracts features from images primarily using convolutional operations and Spatial Pyramid Pooling-Fast (SPPF) [26]. The neck network uses a feature pyramid structure to incorporate different scale features obtained from the backbone network. Obtaining more comprehensive semantic information improves the model’s detection performance. The detection header is responsible for generating target location, species and confidence information. It consists of three output layers at different scales. Each output layer is responsible for detecting target objects of different sizes to improve the model’s ability to detect targets of different scales.

FasterNet - YOLO shows four main improvements in comparison to YOLOv5. First, the backbone network of the YOLOv5 model is improved using a lightweight FasterNet network. Reducing redundant parameters in the backbone network and reducing model complexity while maintaining detection accuracy. Subsequently, the CBS structure in the neck network is replaced with a DBS structure to achieve lightweighting. Afterwards, replace the C3 module in the neck network with a C3STR module from fusion Swin-Transformer. By introducing some discrete parameters of Transformer, the semantic information and feature representation of small targets are enhanced with the help of window self-attention module. Solves the problem of cluttered background of defect images and easy confusion of defect categories. Finally, BiFPN is used for feature fusion. The detector’s adaptability to targets of different scales is improved by retaining more informative features.

### 2.1 FasterNet-based backbone network improvement

MobileNet, ShuffleNet, GhostNet, and other algorithms are often used to detect surface defects in lightweight hot-rolled strips [27–29]. The use of DWConv deep convolution or GConv group convolution has been achieved to lighten the model detection task [30,31]. In the process of reducing the number of network references, as operators often suffer from side effects due to increased memory accesses. In addition, such networks are usually accompanied by additional data operations such as splicing, shuffling and pooling. These operations are critical to the inference speed of lightweight networks. In lightweight networks, inverted residuals and linear bottleneck structures are often used to reduce the overall number of parameters in the network model. However, DWConv in this series of lightweight operations increases the network width, resulting to higher memory accesses. This increases computation and decreases the computational reasoning speed of the network model. It is not conducive to real-time detecting of surface defects on hot-rolled strips, particularly in devices with low computational power.

FasterNet is a new neural network proposed at CVPR 2023 [32]. It possesses superior capabilities in terms of speed, accuracy, and performance compared to other networks like MobileVit, across a diverse variety of devices including CPU, GPU, and ARM processor. FasterNet designs a new partial convolution (PConv). By applying a convolution operation to some of the channels of the input feature map while leaving other channels unchanged [33]. The effect of operators suffering from the side effect of increased memory access in the extraction of spatial features using DWConv and GConv for earlier backbone networks such as MobileNet, ShuffleNet and GhostNet is addressed.

YOLOv5 has a complex backbone network structure, and when deployed on a low computational power device, it detects speeds that do not meet usage requirements. To improve the speed of model detection, the backbone network of YOLOv5 has been lightweighted and improved. The backbone network of YOLOv5 consists mainly of five CBS structures and four C3 structures. Each CBS structure contains of one ordinary convolution, and each C3 structure has at leastfive ordinary convolutions. Each input channel requires ordinary convolutional operations and each channel requires a set of convolutional kernels. This leads to a large number of network parameters and high memory usage. The FasterNet network consists mainly of an embedding layer, a merging layer and a FasterNet block. It has a simple structure and low computational complexity. Improving the backbone network of YOLOv5 by introducing the FasterNet network can effectively reduce the parameters in the backbone network.

PConv uses ordinary convolution for spatial feature extraction on only some of the input channels. Keeping the rest of the channels unchanged at the same time ensures that the inputs and outputs have the same number of channels. The computational complexity of the model is effectively reduced while preserving the spatial information. Pointwise Convolution (PWConv) uses a 1 × 1 convolution kernel for each pixel point of the input and is able to fully utilize the information from all channels.

Assume that h is the height of the feature map, w is the width of the feature map, k is the size of the convolution kernel, c is the number of channels, cp is the number of channels in PConv where the operation is performed, and r is the ratio of $c_{p}$ to c in PConv. GFLOPs for PConv at r = $\frac{c_{p}}{p}=1/4$:


$$h\times w\times k^{2}\times c_{p}^{2}$$

Only 1/16 of an ordinary convolution.

The memory accesses of PConv are as follows:


$$h\times w\times 2c_{p}+k^{2}\times c_{p}^{2}\approx h\times w\times 2c_{p}$$

Only 1/4 of an ordinary convolution. It is evident that PConv significantly decreases the number of GFLOPs and memory accesses in comparison to ordinary convolution. Introducing PConv can reduce the computational complexity of the model.

**Fig 2:**
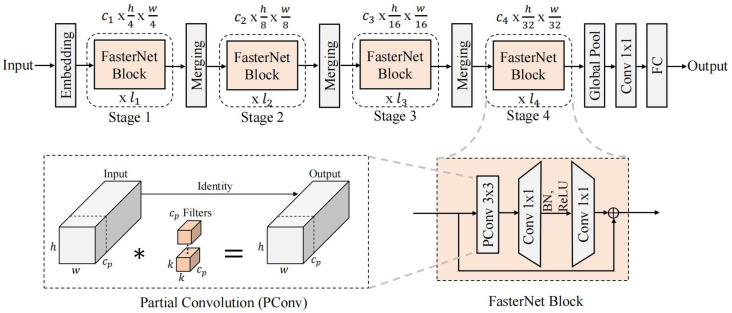
The backbone network improvements based on the FasterNet network.

The FasterNet block consists of one PConv and two PWConvs. BN and activation functions are used only after the intermediate PWConv layer. Lower latency and faster inference are achieved while ensuring feature diversity. The combined structure of PConv and PWConv has a T-shaped effective receptive field on the input feature map. The center location information is given more importance than the regular convolution of the ordinary processing. Combining PConv and PWConv not only reduces computational complexity, but also improves output quality. The backbone network improvements based on the FasterNet network are shown in Fig 2. First, the FasterNet network contains embedding and merging layers to achieve downsampling and increase the number of channels. Second, FasterNet blocks are introduced for feature extraction. Finally, the SPPF structure of YOLOv5 is retained to enhance the model’s ability to perceive targets at different scales. When compared to YOLOv5, the FasterNet-based backbone network has lower computational complexity. At the same time, the information of all channels is fully utilized to maintain a high feature extraction capability.

### 2.2 Improvement of neck networks based on depth-separable convolution

The YOLOv5 neck network’s CBS structure has a large number of ordinary convolutional parameters and uses a lot of memory, leading to slowed model detection. In order to reduce the number of neck network parameters and improve the speed of model detection, the ordinary convolution in the CBS structure is lightened and improved. Depthwise Separable Convolution (DSConv) has a small number of parameters and a small memory usage [34]. The use of DSConv to improve the ordinary convolution in the CBS structure can effectively reduce the number of parameters in the neck network and improve the speed of model detection.

DSConv contains two stages. The first stage is depthwise convolution. This stage performs convolution for each channel of the input using a separate convolution kernel. The second stage is pointwise convolution. This stage adjusts the number of channels using convolution for the results of pointwise convolution.

Assume $D_{F}$ is the input feature size, M is the number of input channels, $D_{H}$ is the output feature size, N is the number of output channels, and $D_{K}$ is the convolution kernel size. The computation of ordinary convolution $C_{1}$ is:


$$C_{1}=M\times D_{K}\times D_{K}\times N\times D_{F}\times D_{F}$$

The computation $C_{2}$ of DSConv is:


$$C_{2}=M\times D_{K}\times D_{K}\times D_{F}\times D_{F}+M\times N\times D_{F}\times D_{F}$$

The ratio of DSConv to ordinary convolutional computation is:


$$\frac{C_{2}}{C_{1}}=\frac{M\times D_{K}\times D_{K}\times D_{F}\times D_{F}+M\times N\times D_{F}\times D_{F}}{M\times D_{K}\times D_{K}\times N\times D_{F}\times D_{F}}$$

From the above equation, it can be seen that the computational volume of DSConv is much less than that of normal convolution.

**Fig 3:**
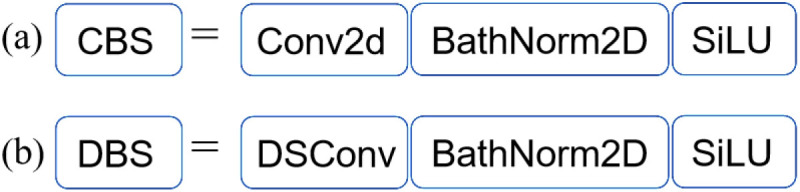
The CBS structure improvement based on DSConv.

The improved structure of the model CBS based on DSConv is shown in Fig 3. Introducing DSConv into the neck network of YOLOv5 reconstructs the ordinary convolution in the CBS structure into depthwise convolution and pointwise convolution. Define this structure as DBS. By reducing the number of parameters and memory usage of the YOLOv5 neck network, the speed of model detection can be improved.

### 2.3 C3STR module incorporating Swin-Transformer

**Fig 4:**
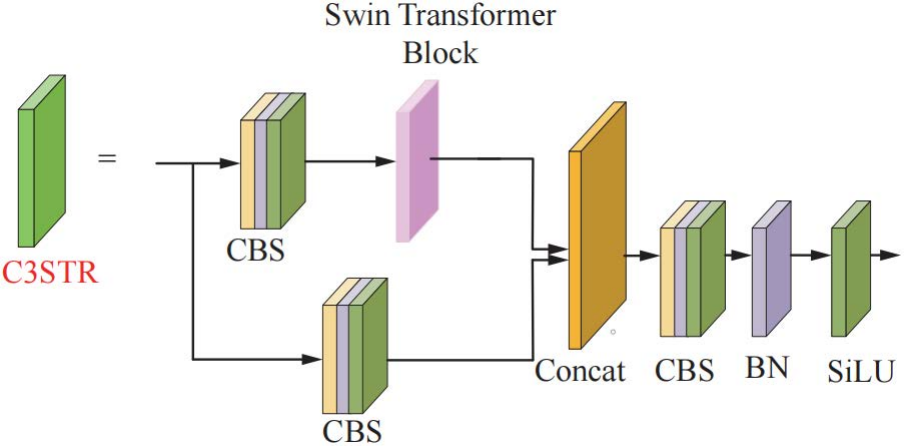
The improved C3STR structure.

With the deepening of the network structure and multiple convolution operations, most of the target feature information that should be available for small targets in the hot-rolled strip surface defect image is lost in the advanced feature map. Therefore, Swin-Transformer is embedded into the C3 convolutional block in the feature fusion section [35]. The window self-attention module improves the semantic information and feature representation of small targets by introducing discrete parameters of the Transformer. The improved C3STR structure is shown in Fig 4.

Swin Transformer Block (STB) mainly consists of Window multi-head self-attention (W-MSA) module and shifted window multi-head self- attention (SW-MSA) module. These two sub-modules significantly reduce the computational complexity compared to the Transformer self-attention MSA sub-module by restricting the computation to windows. The computational complexity of the sub-module is as follows:


$$\Omega_{MSA}=4hwC^{2}+2{\left(hw\right)}^{2}C$$

$$\Omega_{W-MSA/SW-MSA}=4hwC^{2}+2M^{2}hwC$$

Where: Ω denotes the computational complexity; M is a constant (usually set to 7); h, w denote the values of feature height and width, respectively. From equation, the computational complexity of W-MSA and SW-MSA sub-modules is linearly related to h and w, and the computational complexity of MSA module is quadratically related to h and w. Self-attention computation is conducted in the window and then the output is obtained by Multi-layer perceptron (MLP).

The features enter the STB and go to the W-MSA sub-module after Layer Normalization (LN) in the first part. Self-attention calculation based on the moving window through the second part of the SW-MSA sub-module. The final prediction is obtained through performing global average pooling using MLP. where self-attention is calculated as:


$$Attention(Q,K,V)=SoftMax(\frac{QK^{T}}{\sqrt{d}}+B)V$$

Where: SoftMax is the normalized exponential function; Q,  K, and V are the matrices corresponding to Query, Key, and Value, respectively; Query and Key are the feature vectors for calculating the Attention weights, and Value denotes the vector of the input features; d is the vector dimensions of Q and K; and B is the relative position bias matrix.

### 2.4 Using the BiFPN network

Traditional FPNs use a top-down approach to aggregate multi-scale feature elements [36]. However, this unidirectional transmission information transfer method will lose some of the detailed information. PANet is the addition of bottom-up aggregation to FPN to effectively fuse different levels of features. The PAFPN structure used in YOLOv5 combines the advantages of FPN and PANet [37]. Although the limitation of unidirectional information flow of FPN is improved and more efficient feature fusion is realized, the training parameters will be increased accordingly.

**Fig 5:**
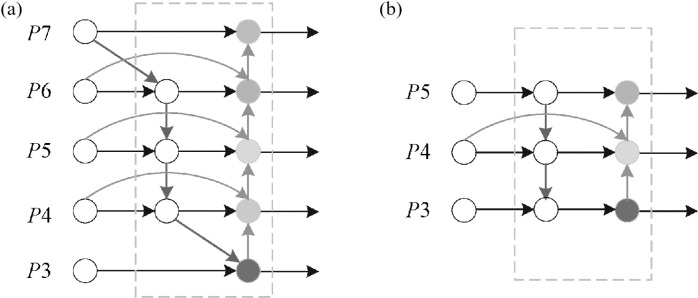
(a) The network structure of BiFPN; (b) The BiFPN network structure used in this paper.

Bidirectional Feature Pyramid Network (BiFPN) applies top-down and bottom-up bi-directional multi-scale feature fusion to aggregate features of different resolutions [38]. Learnable weights are also introduced to learn to update the weights of different input features to improve the detection accuracy. The network structure of BiFPN and the BiFPN network structure used in this paper is shown in Fig 5.

$P_{6}^{td}$ is the intermediate feature of P6 layer and $P_{6}^{in}$ and $P_{6}^{out}$ are the input and output features. The band-weighted feature fusion formula for BiFPN is:


$$P_{6}^{td}=Conv\left(\frac{\omega_{1}\cdot P_{6}^{in}+\omega_{2}\cdot Resize\left(P_{7}^{in}\right)}{\omega_{1}+\omega_{2}+\varepsilon }\right)$$

$$P_{6}^{td}=Conv\left(\frac{\omega_{1}^{\prime }\cdot P_{6}^{in}+{\omega_{2}^{\prime }\cdot P_{6}^{td}+\omega }_{3}^{\prime }\cdot Resize\left(P_{5}^{out}\right)}{\omega_{1}^{\prime }+\omega_{2}^{\prime }+\omega_{3}^{\prime }+\varepsilon }\right)$$

Where: Conv is the corresponding convolution operation; Resize is the up-sampling or down-sampling operation; ω is the corresponding weight of each layer, which is used to distinguish the importance of different features in the process of feature fusion; and ε is a very small non-zero number.

BiFPN networks are capable of conducting multi-level feature fusion across different scales, while also having bi-directional connectivity. The model removes the P3 and P7 layer feature fusion nodes that contribute less to the network to reduce the computational effort and adds an edge to connect the input deterioration outputs. Get deep semantic information and retain more location information without adding more cost. Small target detection accuracy is improved by fusing shallow feature maps. This reduces the cost of computation and storage while increasing detection accuracy.

Hot rolled strips have many surface defects and small targets, such as pitted surface, rolled-in scale, and extreme aspect ratio targets, such as scratch, inclusion. Therefore, this paper uses the BiFPN network principle to add a connection line between the input and output. The P5 and P3 layer fusion points are also retained to preserve more information features. The fusion of defect features at different scales improves the precision of detecting surface defects on hot-rolled strip steel.

## 3 Experiments

### 3.1 Datasets

**Fig 6:**
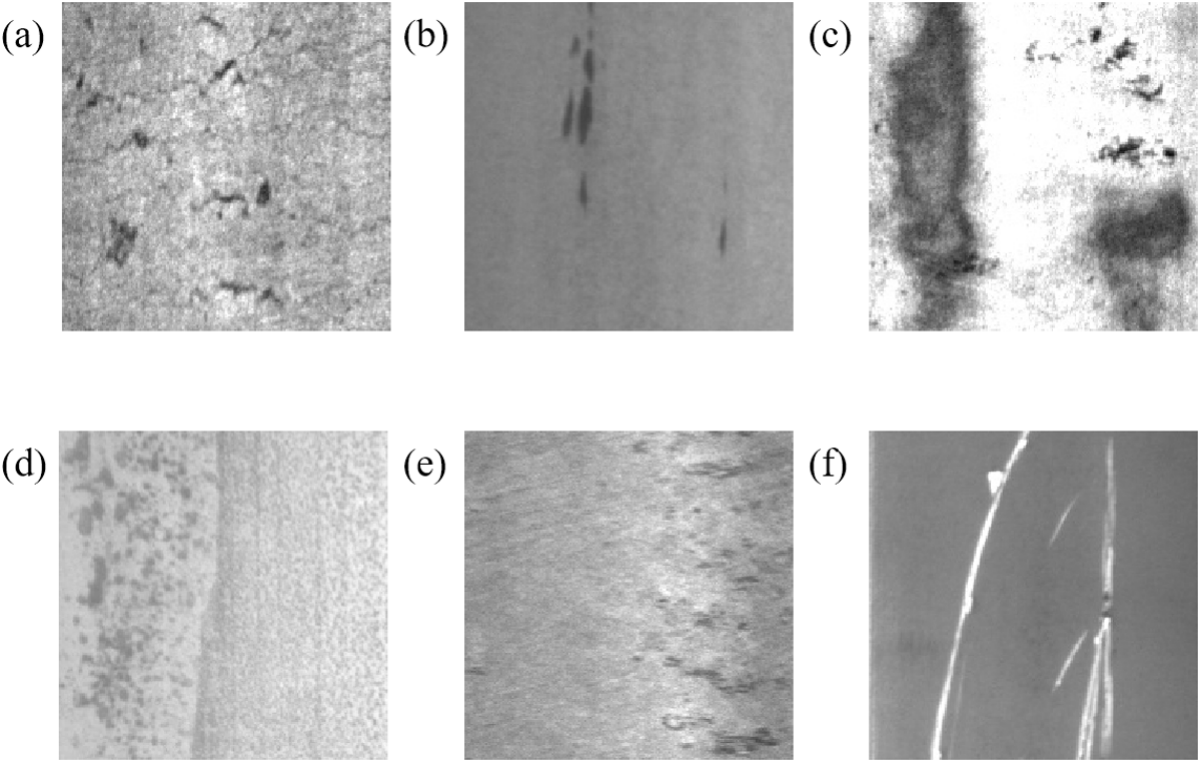
(a) Cr;(b) In;(c) Pa;(d) PS;(e) RS;(f) Sc.

The experiments in this paper use the publicly available dataset NEU-DET (Steel Surface Defects) produced by Northeastern University to train and test the model [39]. The NEU-DET dataset comprises six different types of steel surface defects: Cr (crazing), In (inclusion), Pa (patches), PS (pitted surface), RS (rolled-in scale and scratches), and Sc (scratches). There are 1800 images in total, 300 images for each defect. The training set, validation set, and test set are divided in a ratio of 8:1:1. The six defects of the dataset are shown in Fig 6.

### 2.2 Training Details

The specific configuration of the experimental environment is shown in Table 1.

**Table 1:** Experimental environment configuration.

experimental environment	configuration
CPU	AMD R7 4800H CPU
GPU	NVIDIA GeForce RTX2060 GPU 6 GB
random access memory	16 GB RAM
programming language	Python 3.8.13
deep Learning Framework	PyTorch 1.12.1
CUDA	CUDA 11.7

The parameter settings for this experiment are shown in Table 2.

**Table 2:** Experimental parameter settings.

parameter name	parameter value
training epoch	300
image size	640 × 640
batch_size	16
learning rate	0.01
momentum	0.937
weight decay	0.0005

The training process employs a transfer learning method, where pre-trained weight parameters are loaded on the hot rolled strip surface defect dataset. Throughout the initial three training Epochs, the model’s learning rate gradually increases. Upon the completion of three Epochs, the learning rate will be modified to 0.01. As the Epoch iterates, the learning rate will gradually decrease in order to avoid overfitting the model.

In order to evaluate the overall performance of the model, the experiments used Average Precision (AP), mean Average Precision (mAP) as an index to evaluate the detection effect of the model. AP is the average precision of a single category, which is the region enclosed by the P-R curve. Where P stands for precision and R is recall. TP, FP and FN represent the number of positive samples predicted to be positive cases, the number of negative samples predicted to be positive cases and the number of positive samples predicted to be negative cases, respectively.


$$P=\frac{TP}{TP+FP}$$

$$R=\frac{TP}{TP+FN}$$

$$AP=\int_{0}^{1}P(R)dR$$

$$mAP=\frac{1}{N}\sum_{i=0}^{N}AP(i)$$

In addition to detection accuracy, another important evaluation criterion for object detection tasks is speed, which plays a crucial role in real-time tasks. The metric that usually judges the speed of object detection is frames per second (FPS), i.e., the number of images that can be processed in each second. It is particularly important to note that the calculation of the FPS metric needs to be completed under the same hardware conditions.

## 4 Results and discussions

### 4.1 Ablation study

The paper carries out ablation experiments on the NEU-DET dataset to validate the improved model’s effectiveness in detecting surface defects on steel. For this purpose, the 10 experiments in Table 3 below are carried out, and each set of experiments is tested with the same parameters and network environment. Where experiment 1 is YOLOv5s network without any improvement strategy. Experiments 2, 3, 4, and 5 represent the introduction of FasterNet, DBS, C3STR, and BiFPN, respectively. Experiments 6, 7, and 8 investigate the effects of DBS, C3STR, and BiFPN on the model with the introduction of FasterNet. Experiment 9 investigates the effects of DBS, C3STR and BiFPN on the model.

**Table 3:** Specific parameters of each model and ablation experiments.

Method	Parameters(M)	GFLOPs	mAP(%)	FPS
YOLOv5s(baseline)	7.08	16.5	69.6	52.1
+FasterNet	3.96	7.8	66.3	78.7
+DBS	6.39	15.6	68.9	58.3
+ C3STR	7.29	17.0	73.2	47.5
+BiFPN	7.00	16.4	72.6	51.4
+FasterNet + DBS	3.47	6.9	66.1	84.9
+FasterNet + C3STR	4.16	8.3	69.3	71.2
+FasterNet + BiFPN	3.98	9.2	67.8	78.0
+DBS + C3STR+BiFPN	6.75	16.0	74.5	56
OURS	3.58	7.1	73.9	80.3

The results from the ablation experiments were analysed. Our model parameters decreased by 49.4%, GFLOPs decreased by 57.0%, mAP increased by 6.2%, and FPS increased by 54.1% when compared to the YOLOv5s model. The introduction of FasterNet achieves a more lightweight model and higher FPS. PConv extracts spatial features using ordinary convolution on some of the input channels. Keeping the rest of the channels unchanged at the same time ensures that the inputs and outputs have the same number of channels. The computational complexity of the model is effectively reduced while preserving the spatial information. Lightweight improvement of the ordinary convolution in the CBS structure by using DSConv. It effectively decreases the number of parameters in the neck network and improves the model detection speed. By fusing Swin-Transformer with the C3 module on the neck. The parameters and GFLOPs had a small rise, while the modeled mAP improved significantly by 5.2%. It effectively enhances the model’s ability to deal with the cluttered background of defect images and the easy confusion of defect types. Replacing the PANet in the neck network with a BiFPN increased the model’s mAP by 4.3%. This is because BiFPN networks can perform multi-level feature fusion across scales while having bi-directional connectivity. Gain deep semantic information and retain more positional information. The shallow feature maps are fused to improve the detection accuracy of small targets such as surface defects on hot-rolled steel strip.

### 4.2 Experimental results

Table 4 shows the mAP of our model in comparison to the six defects of YOLOv5. The mAP of Cr, In, Pa, PS, RS, and Sc increased by 8.8%, 2.3%, 5.1%, 8.9%, 17.5%, and 0.7%, respectively. Cr, PS, and RS show significant improvement, whereas the improvement in Sc is not evident. We analysed this because the accuracy of Sc is already high, while the model’s detection of small target defects could be improved.

**Table 4:** The effect of FasterNet—YOLO on 6 types of defects detection.

Method	Cr	In	Pa	PS	RS	Sc
YOLOv5s	36.3	79.2	85.8	77.9	50.4	88.0
OURS	39.5	81.0	90.2	84.8	59.2	88.6

**Fig 7:**
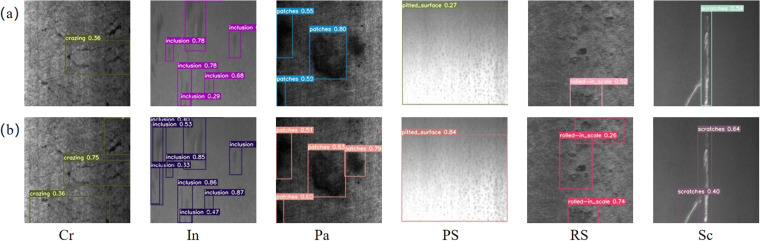
(a) YOLOv5s; (b) FasterNet-YOLO.

Based on the above analysis, the inclusion of FasterNet and DBS has effectively reduced the parameters and GFLOPs of the model. The introduction of Swin-Transformer and BiFPN significantly improves the model’s mAP. Fig 7(a) and 7(b) show some detection results of the YOLOv5s model and our model.

### 4.3 Comparison of different algorithms

In this paper, we choose SSD, Faster R-CNN, YOLOv3, and YOLOv4 to compare their performance to our model. Table 5 shows that our model’s mAP surpasses that of SSD, Faster-RCNN, YOLOv3, and YOLOv4 by a significant margin. The SSD uses a multi-layer feature fusion approach. Smaller targets have limited feature extraction, resulting in poor detection performance. Faster R-CNN is a two-stage detection algorithm. Candidate frames are first generated and then classification and regression operations are performed to get the location and category of the target. So the algorithm has high computation, and the detection speed is difficult to meet the requirements of real-time detection. The YOLOv3 is poor at capturing small targets. The YOLOv4 detection accuracy and speed are both low, making it difficult to deploy. Additionally, the mAP of our model is slightly higher than Retina-Net and YOLOv8. This is due to the fact that most of the current methods for detecting steel surface defects are only good for specific defect categories and lack good applicability to multiple categories of defects. We have made corresponding improvements to address specific needs. Moreover, the FPS of the model is much higher than other models due to the introduction of FasterNet. This indicates that the model is capable of meeting the real-time detection requirements of low computational power platforms.

**Table 5:** Performance of different algorithms.

Types	SSD	Faster-RCNN	Retina-Net	YOLOv3	YOLOv4	YOLOv5	YOLOv8	OURS
Cr	30.4	25.1	45.6	21.5	15.8	36.3	32.7	39.3
In	50.2	65.0	84.3	62.2	72.2	79.2	79.7	81.3
Pa	61.8	75.5	91.0	82.5	87.8	85.8	87.3	90.3
PS	39.0	73.2	74.8	77.2	68.7	77.9	77.9	85.1
RS	70.9	54.4	43.2	30.9	38.8	50.4	54.0	59.3
Sc	51.2	81.2	81.7	83.1	82.2	88.0	89.1	88.0
mAP	51.6	62.4	70.1	59.6	60.9	69.6	70.1	73.9
FPS	31.4	24.0	47.6	60.5	44.9	52.1	66.5	80.3

## 5 Conclusions

In this paper, we propose an algorithm for detection of steel surfaces defects called FsterNet -YOLO based on YOLOv5. The algorithm combines some current techniques in computer vision, including FasterNet, depth-separable convolution, Swin-Transformer, and BiFPN. To address the problems of low detection speed and large number of parameters, the FasterNet network is introduced to reconstruct the backbone network, and the deepwise separable convolution improves the ordinary convolution of YOLOv5’s neck network. To address the problem of cluttered background of defect photos and easy confusion of defect categories. The Swin-Transformer is integrated into the neck network’s C3 module to improve semantic information and feature representation of small targets. In order to improve the adaptability of the detector to targets at different scales. BiFPN is used for feature fusion to retain more informative features. Testing on the NEU-DET dataset reduces model parameters by 49.4%, GFLOPs by 57.0%, and improves mAP by 6.2%. It is demonstrated that the algorithm can detect in real-time with a significant increase in detection speed while slightly improving detection accuracy. In the next research, the model will introduce richer datasets to enhance its generalisation capabilities and make the model better adapted to real-time monitoring in industrial scenarios. In this paper, the experience of processing steel surface defects dataset and designing detection algorithms accumulated in the experiments is hoped to be helpful for more researchers in dealing with steel surface defects.
